# Association between meteorological season and embryo quality in the era of morphokinetics

**DOI:** 10.5935/1518-0557.20250009

**Published:** 2025

**Authors:** Daniela Paes de Almeida Braga, Amanda Setti, Patricia Guilherme, Assumpto Iaconelli Junior, Edson Borges Jr.

**Affiliations:** 1 Fertility Medical Group/FERTGROUP Medicina Reprodutiva, Sao Paulo, Brazil; 2 Instituto Sapientiae - Centro de Estudos e Pesquisa em Reprodução Assistida

**Keywords:** time-lapse microscopy, morphokinetic assessment, season, melatonin

## Abstract

**Objective:**

This study explores the influence of seasonal variations on embryo morphokinetics and the outcomes of intracytoplasmic sperm injection (ICSI) cycles.

**Methods:**

This retrospective cohort study, performed in a private university-affiliated IVF center from March 2019 - March 2023, included 1,292 intracytoplasmic sperm injection cycles (ICSI) cycles and 8,376 injected oocytes. Cycles and were split depending on the season in which oocyte collection was performed: Spring (n=462 cycles), Summer (n=176 cycles), Autumn (n=258 cycles), and Winter (n=396 cycles). Embryos were cultured in a time-lapse imaging (TLI) incubation system, and embryo morphokinetics and laboratory and clinical outcomes were compared between the groups.

**Results:**

A slower morphokinetic development was observed in embryos derived from cycles performed in the winter compared to those from cycles performed in other seasons, while embryos derived from cycles performed in the summer exhibited faster embryo development. Significantly longer time to complete synchronous divisions t8-t5 (s3) and second (cc2, t3-t2) and third cell cycles (cc3, t5-t3) were also observed among embryos derived from winter cycles, whereas embryos formed during summer presented shorter cycles. Embryos from cycles performed during summer exhibited a significantly higher KIDScore compared to those from winter cycles. Significantly higher implantation rate was observed in cycles performed in the summer, followed by those performed in the spring.

**Conclusions:**

These findings suggest a potential influence of seasonal factors on embryo development and implantation success. The study underscores the importance of considering seasonal variations and their potential biological impacts on assisted reproductive technologies.

## INTRODUCTION

The rotation of the earth around its axis, coupled with its orbit around the sun, induces predictable environmental fluctuations on both daily and yearly scales. These rhythmic changes have profoundly influenced evolutionary processes. Consequently, animals manifest the appropriate physiological responses at optimal times. Most species utilize the photoperiod to regulate seasonal transitions in activities such as breeding, fattening, migration, torpor, and hibernation (Dardente *et al.*, 2016).

In mammals, photoperiodic signals are conveyed from the retina to the hypothalamic suprachiasmatic nuclei (SCN), which house the primary circadian oscillator. This master clock orchestrates the synchronization of numerous central and peripheral clocks via neural and endocrine mechanisms. Within the domain of seasonality, the SCN’s daily modulation of the pineal gland plays a crucial role by regulating the patterns of nocturnal melatonin secretion. Consequently, melatonin imparts temporal cues-both diurnal and seasonal-to cells expressing its designated receptors (Karsch *et al.*, 1984; Pevet & Challet, 2011; Simonneaux & Ribelayga, 2003).

Reproductive seasonality in humans has been established, reflecting patterns observed across numerous mammalian species. This phenomenon is evidenced by variations in rates of natural conception and corresponding birth rates (Becker, 1991; Cowgill, 1966; Lam & Miron, 1991; Rojansky *et al.*, 1992; Rosenberg, 1966; Wesselink *et al.*, 2020).

In humans, the impact of seasonal variations on natural conception can be attributed to two primary factors: climatic conditions and temperature influence on male sperm quality (Abdelhamid *et al.*, 2019), and sunlight exposure affecting female ovulation (Gotlieb *et al.*, 2020). Optimal spermatogenesis in males necessitates a testicular temperature that is 2-6°C lower than the overall body temperature. Any increase beyond the physiological temperature of the testes adversely affects spermatogenesis (Liu, 2010; Mieusset & Bujan, 1995).

In vitro fertilization (IVF) involves rigorous control of temperature, humidity, lighting, and other conditions, with considerable efforts made to standardize protocols and laboratory practices to reduce any external impacts on IVF outcomes. Despite these measures, some studies have reported the influence of seasonal variability on IVF results (Braga *et al.*, 2012; Correia *et al.*, 2022; Leathersich *et al.*, 2023; Rojansky *et al.*, 2000). Conversely, some researchers have not identified any climatic or seasonal influence on the outcomes of assisted reproduction cycles (Korkmaz *et al.*, 2023; Liu *et al.*, 2019).

Although the influence of seasonal variation on reproduction has been well-documented and extensively explored in mammals, understanding how seasonal changes affect human and primate reproduction, including the precise underlying mechanisms, remains limited.

Time-lapse imaging (TLI) technology has been employed in IVF laboratories to identify high-quality embryos based on morphokinetic parameters. This technology utilizes a camera attached to a microscope, allowing for the *in situ* assessment of embryos without necessitating their removal from the incubator (Motato *et al.*, 2016; Valera *et al.*, 2022). Consequently, TLI represents a valuable approach for investigating the influence of climatic conditions on embryo development and may serve as a tool to elucidate the mechanisms by which variations in temperature, day length, and sunlight exposure affect embryo quality, if such effects exist. Accordingly, the objective of this study was to assess the impact of meteorological seasons on embryo morphokinetics and the success rates of intracytoplasmic sperm injection (ICSI) cycles.

## MATERIALS AND METHODS

### Patients and experimental design

This retrospective cohort study, performed in a private university-affiliated IVF center from March 2019 - March 2023, included 1,292 intracytoplasmic sperm injection cycles (ICSI) cycles and 8,376 injected oocytes. Cycles and were split depending on the season in which oocyte collection was performed: Spring, n=462 cycles, and 2,801 injected oocytes; Summer n=176 cycles, and 1,040 injected oocytes; Autumn n=258 cycles, and 1,843 injected oocytes; and Winter n=396 cycles, and 2,692 injected oocytes.

Embryos were cultured in a TLI incubation system, and embryo morphokinetics and laboratory and clinical outcomes were compared between the groups.

All patients signed a written informed consent form in which they agreed to share the outcomes of their cycles for research purposes, and the study was approved by the local Institutional Review Board.

### Controlled ovarian stimulation and laboratory procedures

On the third day of the cycle, controlled ovarian stimulation was initiated by the administration of daily doses of r-FSH (300 IU follitropin alpha, Gonal-F, Serono, Geneva, Switzerland or 16 mcg follitropin delta, Rekovelle®, Ferring, Saint-Prex, Switzerland). The r-FSH dose was adjusted according to follicular development, which was monitored by ultrasound scan.

When at least one follicle ≥14 mm was visualized, patients received a subcutaneous injection of 0.25 GnRH antagonist (GnRHa, Cetrotide®; Merck KGaA, Darmstadt, Germany). When three or more follicles attained a mean diameter of ≥ 17 mm and adequate serum estradiol levels were observed, r-FSH and GnRH antagonist administrations were stopped, and final follicular maturation was triggered by the subcutaneous (SC) administration of recombinant human chorionic gonadotropin (r-hCG, Ovidrel®, Merck KGaA, Darmstadt, Germany).

Oocyte recovery was performed 37 hours after the administration of r-hCG through transvaginal follicular aspiration guided by ultrasound, during which the patient was placed under sedation. Oocytes in metaphase II were selected for ICSI.

### Semen analysis and preparation

Semen samples were collected in the laboratory by masturbation. After liquefaction for 30 minutes, semen samples were evaluated for sperm count and motility per the instructions of the count chamber manufacturer (Leja^®^ slide, Gynotec Malden, Nieuw-Vennep, the Netherlands).

Sperm motility was assessed in 100 random spermatozoa and characterized as (i) progressive motility, (ii) nonprogressive motility and (iii) immotile, and the motility was expressed as a percentage.

Sperm morphology was evaluated on air-dried smears that were fixed and stained using the quick-stain technique (Diff-Quick, Quick-Panoptic, Amposta, Spain). A total of 200 sperm cells were characterized as morphologically normal or abnormal, and the final morphology was expressed as a percentage.

Sperm samples were prepared using a two-layered density gradient centrifugation technique (50% and 90% isolate, Irvine Scientific, Santa Ana, CA, USA).

### Intracytoplasmic sperm injection

Intracytoplasmic sperm injection was performed according to Palermo *et al.* (1992). Sperm were selected at 400x magnification using an inverted Nikon Eclipse TE 300 microscope and injected into the oocytes in a microinjection dish prepared with buffered medium (Global w/HEPES, LifeGlobal, Guilford, USA) covered with paraffin oil (Paraffin Oil P.G., LifeGlobal) on an inverted microscope heated stage (37.0°C±0.5°C).

### Embryo culture

Injected oocytes were individually cultured in a 16-well culture dish (Embryoslide, Unisense Fertilitech, Aarhus, Denmark) in 360µl of continuous single-culture media (global^®^ total^®^, LifeGlobal) overlaid with 1.8ml of mineral oil (Paraffin Oil P.G., LifeGlobal) in a TL-monitored incubator (EmbryoScope+, Unisense Fertilitech, Aarhus, Denmark) set at 37°C with an atmosphere of 6% O_2_ and 7.2% CO_2_ until day five of embryo development. A high-definition camera was set up in the incubator to record embryo images in eleven focal planes every 10 minutes. Recorded kinetic markers were time to pronuclei appearance (tPNa) and fading (tPNf); time to two (t2), three (t3), four (t4), five (t5), six (t6), seven (t7), and eight cells (t8); timing to morulation (tM); time to start of blastulation (tSB); and time to blastulation (tB). The durations of the second (cc2, t3-t2) and third cell cycles (cc3, t5-t3) and the time to complete synchronous divisions (t2-tPNf (s1), t4-t3 (s2) and t8-t5 (s3)) were calculated. Data generated from EmbryoScope+ were analyzed using EmbryoViewer software (Vitrolife^®^, Copenhagen, Denmark). The incidences of multinucleation at the 2- and 4-cell stages and of abnormal cleavage patterns (direct or reverse cleavage) were recorded for each embryo.

### Clinical follow-up

Embryo transfer was performed on Day 5 of embryo development, and one or two embryos were transferred per patient. The decision regarding the number of embryos to be transferred considered the patients’ age and embryo quality.

Women with a positive pregnancy test, performed 10 days after embryo transfer, had a transvaginal ultrasound scan 2 weeks later. Clinical pregnancy was diagnosed upon detection of fetal heartbeat. The pregnancy rate was calculated per embryo transfer. The implantation rate was calculated as the number of gestational sacs with fetal heartbeats divided by the number of transferred embryos. Miscarriage was defined as clinical pregnancy loss before 20 weeks. The cancellation rate was defined by the number of initiated cycles divided by the number of cycles in which there was no transfer due to the absence of viable embryos for transfer.

### Data analysis and statistics

The primary outcome measure was tB since it is the most advanced key stage of embryonic development recorded in our center. Post hoc power analysis was calculated, given an α of 5%, a sample size of 554 embryos that reached the blastocyst stage at Day 5 of development, and an effect size for tB. The achieved power was greater than 80%. The calculation was performed using the Hotelling Lawley Trace test in the GLIMMPSE app for multilevel data (Kreidler *et al.*, 2013), which accounted for the correlation between embryos from the same cycle.

Morphokinetics and clinical ICSI outcomes were compared between the groups using generalized linear models, followed by the Bonferroni post hoc test adjusted for potential confounders, such as maternal and paternal ages, number of transferred embryos, and endometrial thickness.

A random effect was added to account for the correlation between the embryos within the same cycle, with a linear distribution for morphokinetic data in hours (h) and Known Implantation Data Score (KIDScore) ranking. For clinical outcomes, which were based on a single observation per couple, generalized linear models were used without random effects with a linear distribution for implantation rate and a binomial distribution for clinical pregnancy, miscarriage, and live birth rates.

The results are expressed as percentages or means ± standard errors (SEs) and p values. *p*<0.05 was considered statistically significant. Data analysis was conducted using the Statistical Package for the Social Sciences (SPSS) 21 (IBM, New York, NY, USA).

## RESULTS

The demographic data categorized by seasonal groups are presented in Table 1. A significant increase in paternal age was observed in patients undergoing cycles in autumn compared to the other seasons. However, the distribution of the other variables was comparable across the groups.

**Table 1:** Demographic data of patients undergoing intracytoplasmic sperm injection

Demographic data	Winter	Autumn	Spring	Summer	*p*
	n:369	n:258	n:462	n:176	
Female Age (years)	37.4±1.9	37.5±2.3	37.9±1.7	37.0±2.8	0.052
BMI (kg/m2)	24.3±18.4	24.3±23.0	25.1±17.0	23.8±27,2	0.610
Male Age (years)	39.3±3.0a	39.4±3.8a	40.8±2.8b	39.0±4.6a	< 0.01
Total dose of FSH (IU)	2622.9±47.8	2678.6±65.2	2663.1±46.7	2699.2±79.1	0.821
Serum oestradiol (pg/mL)	2348.6±182.4	2017.0±261.6	2066.6±204.6	2358.4±266.8	0.589
Number of follicles	13.9±0.52	14.2±0.64	12.9±0.47	13.3±0.77	0.340
Number of retrieved oocytes	10.0±4.0	10.3±5.1	9.4±3.7	9.8±6.1	0.433
Percentage of retrieved MII	7.5±3.1	7.6±3.9	6.9±2.9	7.1±4.7	0.377

Values expressed as average±standard errors. Different subscripts within the same line indicate statistically significant difference. n: Number of patients.

Lower tPNa, tM, and tSB were observed in embryos derived from cycles performed in the winter compared to those from cycles performed in other seasons. On the other side, embryos derived from cycles performed in the summer exhibited faster tPNa, tM, and tSB than those from other seasons (Table 2).

**Table 2:** Demographic data of patients undergoing intracytoplasmic sperm injection

Morphokinetic parameter (h)	Winter	Autumn	Spring	Summer	*p*
	n:2,692	n:1,843	n:2,801	n:1,040	
tPNa	6.8±0.7^a^	6.4±0.8^b^	6.8±0.6^a^	6.3±0.1^b^	**< 0.01**
tPNf	24.2±1.6	24.4±1.2	24.2±1.0	24.0±1.0	0.089
t2	26.7±1.7	26.9±1.3	26.9±1.1	26.5±1.1	0.059
t3	37.4±2.0	37.7±1.5	37.4±1.3	37.2±1.3	0.124
t4	39.1±2.1	39.3±1.5	39.2±1.3	38.9±1.3	0.362
t5	49.9±3.0	50.0±2.2	49.9±1.9	49.8±1.9	0.943
t6	53.0±3.0	52.6±2.2	52.7±1.9	52.8±1.9	0.774
t7	55.9±3.3	55.2±2.4	55.6±2.1	55.4±2.1	0.321
t8	59.6±3.8	58.7±2.8	59.3±2.4	58.9±24.1	0.190
tM	90.1±4.2^a^	88.6±3.1^b^	88.8±2.6^b^	88.1±2.5^b^	**> 0.01**
tSB	102.5±4.1^a^	100.7±3.0^b^	100.9±2.5^b^	99.9±2.5^c^	**> 0.01**
tB	111.4±4.8	110.2±3.5	110.6±2.9	110.0±2.8	0.08
s1	2.3±0.9	2.2±0.7	2.3±0.6	2.2±0.6	0.621
s2	1.4±0.9	1.3±0.7	1.3±0.6	1.3±0.6	0.381
s3	7.7±2.6^a^	7.1±1.9^a,b^	6.8±1.6^b^	6.7±1.6^b^	**> 0.01**
cc2	8.9±1.7^a^	8.8±1.3^a^	8.2±1.0^b^	8.0±1.0^b^	**> 0.01**
cc3	10.2±2.3^a^	10.1±1.7 ^a^	9.1±1.4 ^b^	9.5±1.4^b^	**> 0.01**
KIDScore	4.3±0.9^a^	4.6±0.7^a,b^	4.6±0.6^a,b^	4.8±0.6^b^	**> 0.01**

Significantly longer s3, cc2, and cc3 were also observed among embryos derived from winter cycles, whereas embryos formed during summer presented shorter cycles (Table 2).

Moreover, embryos from cycles performed during summer exhibited a significantly higher KIDScore compared to those from winter cycles (Table 2).

Regarding ICSI outcomes, although a significantly lower fertilization rate was observed in cycles performed in the summer and spring compared to those performed in the winter and autumn, a significantly higher implantation rate was observed in cycles performed in the summer, followed by those performed in the spring. In contrast, cycles performed in the autumn and winter exhibited lower implantation rates (Table 3).

**Table 3:** Comparison of ICSI outcomes between the seasons

ICSI outcomes	Winter	Autumn	Spring	Summer	*p*
	n:369	n:258	n:462	n:176	
Fertilization rate (%)	85.0±1.3^a^	84.7±1.6^a^	76.6±1.2^b^	75.8±1.9^b^	< 0.01
Blastocyst formation rate	58.0±1.6	56.2±2.0	53.7±1.5	55.8±2.4	0.287
Pregnancy rate (%)	31.1±4.8	44.0±7.0	31.3±5.1	50.0±7.7	0.092
Implantation rate	21.9±4.9^a^	31.0±7.8^b^	24.0±5.4^c^	39.3±9.6^d^	< 0.01

Note: Values are means±standard errors, unless otherwise noted. Different subscripts within the same line indicate statistically significant difference. n: Number of patients.

Note: Values are means±standard errors, unless otherwise noted. H - hours, tPNa - time to pronuclei appearance, tPNf - time to pronuclei fading, t2 - time to two cells, t3 - time to three cells, t4 - time to four cells, t5 - time to five cells, t6 - time to six cells, t7 - time to seven cells, t8 - time to eight cells, tSB - time to start of blastulation, tB - time to blastulation, s1 - time to complete t2-tPNf synchronous divisions, s2 - time to complete t4-t3 synchronous divisions, s3 - time to complete t8-t5 synchronous divisions, cc2 - duration of the second cell cycle (t3-t2), cc3 - duration of third cell cycle (t5-t3). Different subscripts within the same line indicate statistically significant difference. n: Embryos.

## DISCUSSION

The association between seasonal variation and assisted reproduction results has not yet been clarified, and conflicting reports have been published. While some authors argue that the season of the year does not impact the success rates of assisted reproductive cycles (Carlsson Humla *et al.*, 2022; Du *et al.*, 2023; Kirshenbaum *et al.*, 2018; Liu *et al.*, 2019), other studies have shown differences in fertilization rates (Braga *et al.*, 2012; Mehrafza *et al.*, 2020; Wood *et al.*, 2006), embryo quality (Chu *et al.*, 2022; Mehrafza *et al.*, 2020), pregnancy rate (Mehrafza *et al.*, 2020; Wood *et al.*, 2006) and livebirth (Mehrafza *et al.*, 2020).

Despite the discrepancy in assisted reproduction studies, when it comes to natural cycles it has been demonstrated that seasonality plays a significant role in influencing fertility in mammals, including humans. As an example, seasonal variations in ovulation frequency, spermatogenic activity, and gamete quality are observed in farm mammals These variations are governed by endogenous circannual rhythms that are synchronized by light and melatonin (Chemineau *et al.*, 2007, 2008).

To investigate the potential effects of light exposure on the quality of embryos derived from ICSI cycles, the present study utilized a non-invasive TLI system. This system allows for continuous monitoring of embryos, facilitating the study of seasonal impacts on embryo morphokinetics.

Our study found that embryos derived from cycles performed in winter showed slower progression of specific morphokinetic milestones compared to those from other seasons. Additionally, embryos derived from cycles conducted in summer showed notably elevated KIDScore in contrast to those originating from winter cycle.

It has been previously demonstrated that embryos with specific morphokinetic parameters in TLI systems show improved implantation rates, indicating a correlation between embryo speed and successful outcomes. Research indicates that embryos with a shorter interval time to morulation and starting blastulation have significantly higher implantation rates compared to those with longer timeframes (Soukhov *et al.*, 2022), what corroborates with our findings.

Time-lapse imaging has been proved to enhance the efficiency of IVF by enabling the selection of embryos with optimal developmental potential for successful implantation and pregnancy outcomes. Therefore, embryos exhibiting slower cell divisions and those with lower KIDscores ideally should not have been selected for transfer. Consequently, we shouldn’t have observed differences in implantation rates based on the season when embryos were cultured using the time-lapse system. However, depending on the number and quality of embryos, selecting faster-developing embryos, with higher scores for transfer may not always be feasible due to limited availability of high-quality embryos.

In contrast to our findings, Chu *et al.* (2022) found that the high-quality embryo rate was higher in autumn and winter compared to spring and summer. This suggests that cooler temperatures during ovulation induction may positively impact embryo quality. Another study indicated that the season at the time of oocyte retrieval did not significantly affect live birth rates, suggesting that embryo quality might not be directly influenced by the season (Leathersich *et al.*, 2023).

Indeed, while some evidence points to seasonal variations affecting embryo quality and pregnancy outcomes, other studies find minimal impact, indicating that further research is needed to clarify these associations. The influence of external factors like temperature and sunshine hours may also play a role, but their exact impact remains uncertain.

The reason why embryos produced during summer exhibit faster development and higher implantation rates is unknown; however, some hypotheses can be proposed. Serum vitamin D levels, which are influenced by sun exposure, might affect human natural conception rates. Some studies have indicated a role of vitamin D in reproductive physiology (Malloy *et al.*, 2009), with some reporting an association between vitamin D sufficiency and improved success rates following IVF (Iliuta *et al.*, 2022).

Another hypothesis concerns the synthesis and secretion of melatonin. Melatonin secretion is typically associated with darkness and the circadian night (Qian *et al.*, 2022). However, studies have shown that exposure to daylight can also influence melatonin levels. A previous study has indicated that daylight exposure can increase nocturnal melatonin secretion, even in uncontrolled daily life settings (Obayashi *et al.*, 2012).

Melatonin, plays a crucial role in human fertility by affecting various aspects of reproductive physiology (Gelen *et al.*, 2022). Tamura *et al.* (2022) have shown that melatonin can improve assisted reproduction cycles by increasing the fertilization rate and blastocyst formation rate. Apparently, melatonin alters the granulosa cells transcriptome and, thus, their functions, and this could improve the oocyte quality (Tamura *et al.*, 2022). Furthermore, embryo culture with melatonin has been found to significantly improve embryo development and clinical outcomes in patients with repeated implantation failure, leading to higher fertilization, cleavage, and blastocyst rates, as well as increased implantation and pregnancy rates (Zhu *et al.*, 2022). Seasonal changes in melatonin secretion also synchronize reproductive activity with variations in day length, highlighting its role in regulating human reproduction (Srinivasan *et al.*, 2009).

The clinical significance of these results lies in their potential to inform and optimize ART practices and patient counseling. The observed seasonal variations in embryo morphokinetics and implantation rates suggest that the timing of oocyte retrieval may influence outcomes. Understanding these seasonal effects could lead to tailored approaches in ART, such as adjusting laboratory protocols to mimic the more favorable conditions of certain seasons or scheduling cycles based on individual patient characteristics and seasonal trends.

A major limitation of our study is its small sample size. However, few studies have focused on analyzing the seasonal association with outcomes in fresh IVF/ICSI cycles where embryos were cultured in TLI incubators. To the best of our knowledge, this is the first study to evaluate the seasonal effect on embryo morphokinetics.

In conclusion, our findings demonstrate that embryos derived from winter cycles show slower progression of morphokinetic milestones, while those from summer cycles exhibit faster development and higher KIDScore, suggesting a potential influence of seasonal factors on embryo development and implantation. The study underscores the importance of considering seasonal variations and their potential biological impacts on ART. Further research is needed to elucidate the underlying mechanisms and to optimize treatment protocols to enhance reproductive outcomes across different seasons.
